# Adsorption of Pyrethroids in Water by Calcined Shell Powder: Preparation, Characterization, and Mechanistic Analysis

**DOI:** 10.3390/ma16072802

**Published:** 2023-03-31

**Authors:** Xiaohan Ma, Siyuan Tao, Shiqian Fu, Huicheng Yang, Bangchu Lin, Yongjiang Lou, Yongyong Li

**Affiliations:** 1 Key Laboratory of Food Deep Processing Technology of Animal Protein of Zhejiang Province, College of Food and Pharmaceutical Sciences, Ningbo University, Ningbo 315800, China; 2 Zhejiang-Malaysia Joint Research Laboratory for Agricultural Product Processing and Nutrition, College of Food and Pharmaceutical Sciences, Ningbo University, Ningbo 315800, China; 3 Hangzhou Yuhang Food and Drug Monitoring & Testing Center, Hangzhou 311112, China; 4 Zhejiang Marine Development Research Institute, Zhoushan 316021, China; 5 Zhejiang Yulin Technology Co., Ltd., Ningbo 315021, China

**Keywords:** adsorption, Box–Behnken design, calcined shell powder, pyrethroid

## Abstract

Pyrethroids are common contaminants in water bodies. In this study, an efficient mussel shell-based adsorbent was prepared, the effects of factors (calcination temperature, calcination time, and sieved particle size) on the pyrethroid adsorption capacity from calcined shell powder were investigated via Box–Behnken design, and the prediction results of the model were verified. By characterizing (scanning electron microscopy, X-ray diffraction, Fourier infrared spectroscopy, and Brunauer–Emmett–Teller measurements) the adsorbent before and after the optimized preparation process, the results showed that calcined shell powder had a loose and porous structure, and the main component of the shell powder under optimized condition was calcium oxide. The adsorption mechanism was also investigated, and the analysis of adsorption data showed that the Langmuir, pseudo second-order, and intra-particle diffusion models were more suitable for describing the adsorption process. The adsorbent had good adsorption potential for pyrethroids, the adsorption capacity of the two pesticides was 1.05 and 1.79 mg/g, and the removal efficiency was over 40 and 70% at the maximum initial concentration, respectively.

## 1. Introduction

Compared to carbamate and organophosphorus pesticides, synthetic pyrethroids are a class of compounds that are widely used for pest control because of their strong insecticidal ability and lower mammalian toxicity, and they are often present in large quantities in environmental water bodies [1,2]. Substantial evidence shows that pyrethroid insecticides are associated with damage to multiple systems in humans, including the nervous, digestive, and reproductive systems [3,4]. Chronic exposure to pyrethroids in pregnant and lactating females can increase the risk of toxicity or death in the offspring [5]. In addition, pyrethroids have significant lethal effects on aquatic species, and the toxicity of pyrethroids to fish is approximately 1000-fold higher than that of mammals and birds [6]. Therefore, the effective reduction of pyrethroid residues in environmental water bodies has positive implications.

For environmental pollutants, there are several commonly used removal methods, such as physical adsorption [7,8], solvent extraction [9], and biodegradation [10], among which physical adsorption has a greater advantage for removing pesticide residues from water bodies. A good physical adsorbent requires low expense, high adsorption capacity, environmental friendliness, and sustainability [11]. Natural inorganic mineral products have been widely studied as adsorbents. Mussels are a common economic shellfish in the southeastern coastal region of China, and they are consumed in large quantities every year. Along with the economic growth brought about by huge consumption, a large amount of untreated shell waste is abandoned in the natural environment. For example, in Zhoushan, the home of mussels in China, the total annual mussel production is nearly 100,000 tons, of which the amount of discarded shells is more than 60,000 tons [12]. The long-term accumulation of untreated shell waste not only easily generates microorganisms and emits an odor, but also occupies a lot of land resources and causes environmental pollution [13]; therefore, effective use of shell resources is an important issue. The main component of mussel shells is carbonate, which is chemically stable, inexpensive, easy to obtain, has a multilayer porous material structure, and has been widely used as an adsorbent material in recent years because of its good adsorption capacity [14,15]. Susana et al. studied the adsorption capacity of mussels for mercury and showed that calcined mussel shells have a high adsorption efficiency for mercury, and phosphate promotes this process [16]. Peinemann et al. successfully removed phosphate from a lactic acid-containing fermentation broth using a shell powder, and the phosphate removal efficiency was as high as 95% after 2 h [17]. Notably, most studies on the adsorption of environmental pollutants have focused on metal ions [18,19], dyes [20,21], and pesticides [22], and much progress has been made in the study of conventional pesticide adsorption, while reports on the adsorption of newer generations of insecticides such as pyrethroids are rare.

In this study, discarded mussel shells were crushed and calcined to make a porous adsorbent for bifenthrin and cypermethrin, which are the most common class of pyrethroids in environmental water bodies [6,23]. Response surface methodology (RSM) was used to optimize the calcination process of the mussel shell powder. The adsorbent under optimal calcination conditions was characterized via X-ray diffraction, scanning electron microscopy, Fourier infrared spectroscopy, and Brunauer–Emmett–Teller measurements. In addition, the adsorption mechanism was analyzed to elucidate the adsorption process, and variables on the adsorption capacity of pesticides were also involved, these results will provide a future reference for the removal of pyrethroid residues from the environment.

## 2. Materials and Methods

### 2.1. Materials and Reagents

Mussel shells were obtained from Shengsi, Zhoushan, China (122°75′ E, 30°71′ N). Bifenthrin and cypermethrin were purchased from HaoLiTe Biopesticide Co., Ltd. (Qingdao, China). Hydrochloric acid (HCl, A.R. grade, 36–38%) and sodium chloride (NaCl, A.R. grade, ≥99.5%) were purchased from Shanghai Sinopharm Chemical Reagent Co., Ltd. (Shanghai, China). Acetonitrile (HPLC. grade, ≥99.9%) and n-hexane (HPLC. grade, ≥99.9%) were purchased from Shanghai Macklin Biochemical Technology Co., Ltd. (Shanghai, China). Unless otherwise specified, all the water used for the experiments was ultrapure.

### 2.2. Equipment and Characterization

A tube furnace (Shuoguang SGL-1200, Shanghai, China) was used for calcining shell powder. Pyrethroid adsorption capacity was analyzed via gas chromatography (GC; Agilent 19091J, Santa Clara, CA, USA); the conditions were: DB-5 column (30 m × 320 μm × 0.25 μm); injection port temperature: 250 °C; carrier gas: N_2_ (99.99%); detector temperature: 280 °C; carrier gas flow rate: 1 mL/min; no splitting. Scanning electron microscope (SEM; HITACHIS 3400-N, Tokyo, Japan) was used to observe the microstructure and surface morphology of the sample; pictures were taken at an accelerating voltage of 15 kV. X-ray diffraction (XRD; Bruker D8 Advance, Karlsruhe, Germany) was used to analyze the phase composition and crystal structure of the sample; radiation source was CuKα; step size: 0.02°; scanning speed: 0.2 s/step; wavelength λ: 0.15406 nm; voltage: 40 kV; current: 40 mA; 2θ range: 5–90°. Fourier Transform Infrared Spectrometer (FT-IR; JASCO FT/IR-4700, Tokyo, Japan) was used to record the IR absorption spectra of the sample and analyze their functional groups; scanning speed: 16 times/s; resolution: 4 cm^−1^. Brunauer–Emmett–Teller analyzer (BET; Micromeritics ASAP-2460, Norcross, GA, USA) was used to analyze the changes in the specific surface area and pore distribution before and after calcination of the materials. Zetapotential analyzer (Anton paar; SurPASS 3; Graz, Austria) was used to measure the zeta potential of the calcined mussel shell powder at different pH.

### 2.3. Preparation of Calcined Shell Powder

Mussel shells were soaked in 0.5% HCl for 2.0 h and then washed with ultrapure water to neutral. The washed mussel shells were dried in a blast dryer till a constant weight was reached and then crushed using an ultra-micro crusher to obtain a shell powder. The shell powder was sieved with a standard sieve, then heated to the set temperature in a tube furnace at 5 °C per minute, and maintained at the highest temperature for a while. After calcination, the sample was cooled to room temperature and passed through the standard sieve again to obtain calcined shell powder. During the calcination process, the highest temperature set was considered the calcination temperature, the time maintained at this temperature was considered the calcination time, and the sieved particle size of the sample is expressed by the mesh of the sieve.

### 2.4. Optimization of the Preparation Process of Calcined Shell Powder

#### 2.4.1. Adsorption Experiment Steps

Bifenthrin and cypermethrin were diluted with acetonitrile to prepare a mixed stock solution (1.0 mg/mL), 0.2 mL mixed stock solution was added to 20 mL water, then 0.1 g calcined shell powder was added to pyrethroid aqueous solution and this stood for 0.5 h. After standing, 10.0 mL supernatant and 5.0 g NaCl were added to 25.0 mL acetonitrile. The mixture was placed in a high-speed centrifuge at 8000 rpm for 2.0 min, and after the mixed solution was completely layered, 5.0 mL of the supernatant was transferred to another centrifuge tube, and nitrogen was blown near dry at 50 °C in a water bath. After nitrogen blowing, the residual solid was re-dissolved in 2.0 mL n-hexane solution, fully shaken, and filtered through 0.22 μm organic system filter membranes before GC analysis. The removal efficiency and adsorption capacity of pyrethroids were calculated with Equations (1) and (2).

(1) $$R=\frac{C_{0}-C_{e}}{C_{0}}\times 100\%$$

(2) $$q_{e}=\frac{\left(C_{0}-C_{e}\right)V}{m}$$

where *C*_0_ (mg/L) and *C_e_* (mg/L) denote the initial and equilibrium concentrations of pyrethroids in solution, respectively, *V* (L) is the volume of the pyrethroid solution, *m* (g) is the mass of adsorbent used, *R* (%) is the removal efficiency of pyrethroids, and *q_e_* (mg/g) denotes the capacity of pyrethroids adsorbed by the adsorbent.

#### 2.4.2. Single-Factor Experiments

To explore the effects of calcination temperature on pyrethroid adsorption capacity, 1.0 g shell powder through 300 mesh sieve was heated in a tube furnace to 550, 650, 750, 850, 950, and 1050 °C, and cooled to room temperature after 3.0 h to determine the pyrethroid adsorption capacity. To investigate the effects of calcination time on pyrethroid adsorption, 1.0 g shell powder through 300 mesh sieve was heated to 850 °C in a tube furnace and then thermostated for 0.5, 1.0, 1.5, 2.0, 2.5, and 3.0 h, and cooled to room temperature to determine the pyrethroid adsorption capacity. To investigate the effects of sieved particle size on pyrethroid adsorption, 1.0 g shell powder was sieved through 150, 200, 250, 300, 350, and 400 mesh sieves, then heated to 850 °C in a tube furnace, and thermostatted for 2.5 h; the capacity of pyrethroid adsorption was measured after cooling to room temperature.

#### 2.4.3. Box–Behnken Design

To investigate the effects of the main factors in the preparation of calcined shell powder on pyrethroid adsorption capacity, Box–Behnken design with 17 groups of experiments was designed, calcination temperature, calcination time, and sieved particle size were selected as the independent variables of the experiments, and the adsorption capacity of the two pyrethroids was taken as response values. The sample obtained under optimal calcination conditions was denoted Optimal Calcined Shell Powder (OCSP), and the shell powder before calcination was denoted Un-calcined Shell Powder (USP). In order to investigate the difference in the capacity of pyrethroid adsorption by shell powder before and after optimal optimization conditions, 0.1 g of USP and OCSP were added to the two pyrethroid solutions, and their pesticide adsorption capacities were compared; each group of experiments was repeated three times and the results were averaged.

### 2.5. Batch Adsorption Experiment

#### 2.5.1. Adsorption Experiments

Unless stated otherwise, the pyrethroid solutions mentioned in this experiment refer to a mixture of bifenthrin and cypermethrin in equal proportions. In order to investigate the effects of reaction time on adsorption behavior, 0.1 g OCSP was added to 20 mL of pyrethroid aqueous solution with a concentration of 10 mg/L, and the pyrethroid adsorption capacity was analyzed at time intervals of 0.5, 2, 5, 10, 15, 20, 25, 30, 35, and 40 min. To investigate the effects of the initial concentration of pyrethroids on the adsorption behavior, 0.1 g OCSP was added to 20 mL of pyrethroid aqueous solution with pesticide initial concentrations of 0.5, 1, 2, 4, 6, 8, and 10 mg/L, respectively; the capacity of pyrethroids adsorption was analyzed after 0.5 h. To investigate the effects of adsorbent dosages on the adsorption behavior, different dosages of OCSP (5, 7.5, 10, 12.5, and 15 g/L) were added separately to 20 mL of pyrethroid aqueous solution with a concentration of 10 mg/L; reaction time was the same as the adsorption isotherm experiment. For the purpose of exploring the effects of pH on the adsorption behavior, 0.1 g OCSP was added to 20 mL of pyrethroid aqueous solution with a concentration of 10 mg/L, and the solution pH values were 6.0, 7.0, 8.0, 9.0, and 10.0, respectively; other experimental conditions remain unchanged (see Section 2.3). To explore the effects of NaCl salinity on the adsorption behavior, 0.1 g OCSP was added to 20 mL of pyrethroid aqueous solution with a concentration of 10 mg/L, and the NaCl salinity of the solution ranges from 0 to 4%; the capacity of pyrethroid adsorption was analyzed after 0.5 h.

#### 2.5.2. Adsorption Kinetic Model

Adsorption kinetics is usually used to analyze the adsorption efficiency of adsorbents, mainly involving the relationship between the action time of the adsorbent and its adsorption capacity [24]. In this study, the pseudo first-order (Equation (3)), pseudo second-order (Equation (4)), and intra-particle diffusion model (Equation (5)) were used to describe the adsorption process by OCSP.

(3) $$\ln\left(q_{e}-q_{t}\right)=\ln\left(q_{e}\right)-\frac{k_{1}}{2.303}t$$

(4) $$\frac{t}{q_{t}}=\frac{1}{k_{2}q_{e}^{2}}+\frac{1}{q_{e}}t\;$$

(5) $$q_{t}=k_{pi}t^{1/2}+C$$

where *q_e_* (mg/g) and *q_t_* (mg/g) are the adsorption capacity of the adsorbent at adsorption equilibrium and time t; *k*_1_ (min^−1^) and *k*_2_ (g/mg·min) are the pseudo first-order and pseudo second-order model constants, respectively; *k_pi_* (mg/g·min^1/2^) is the rate constant of the intra-particle diffusion model; and *C* is the relevant constant.

#### 2.5.3. Adsorption Isotherm Model

Adsorption isotherms could be used to analyze the interaction between the adsorbent and the adsorbed materials at an equilibrium state [20]. Langmuir model (Equation (6)) is based on the adsorbate being monolayer and uniformly adsorbed on the surface of the adsorbent, and there exists no interaction between the adsorbates. The empirical equation of the Freundlich model (Equation (7)) is based on nonideal adsorption on heterogeneous surfaces [24].

(6) $$q_{e}=\frac{q_{m}K_{L}C_{e}}{1+K_{L}C_{e}}$$

(7) $$q_{e}=K_{F}C_{e}^{n}$$

where *q_m_* (mg/g) is the maximum adsorption capacity of the adsorbent, *K_L_* (L/mg) is the Langmuir model constant related to the adsorption rate, *K_F_*, and *n* are Freundlich model constants.

### 2.6. Reusability Studies

To investigate the reusability of adsorbent, the calcined shell powder was filtered out from the pyrethroid solution after the adsorption process, then dried to constant weight at 105 °C in a blast drying oven. Afterward, the pesticide adsorption and drying steps of calcined shell powder were repeated, and the capacity of pyrethroids adsorbed was also measured each time.

### 2.7. Data Analysis

Design Expert 10.0.1 was used for experimental data acquisition and analysis of RSM. Origin 2019b and SPSS 25.0 were used to establish the adsorption model and analyze related data.

## 3. Results and Discussion

### 3.1. Analysis of the Response Surface

Based on the results of the single-factor experiment (Figure S1), the factors and levels (Table S1) for optimization experiments were determined. The model adsorption equations of bifenthrin and cypermethrin are shown in Equations (8) and (9).

Y1 = 0.86 + 0.094 A − 0.005 B + 0.024 C + 0.012 AB − 0.13 AC − 0.023 BC − 0.26 A^2^ − 0.065 B^2^ − 0.33 C^2^ (8)

Y2 = 1.56 + 0.14 A + 0.04 B + 0.15 C − 0.011 AB − 0.20 AC + 0.0062 BC − 0.44 A^2^ – 0.043 B^2^ − 0.64 C^2^ (9)

where Y1 (mg/g) and Y2 (mg/g) refer to the adsorption capacity of bifenthrin and cypermethrin; A, B, and C are the coded values of calcination temperature, calcination time, and sieved particle size.

The results of the response surface optimization experiments and the ANOVA analysis of the model are shown in Tables S2–S4. From the ANOVA analysis, the linear and square effects of calcination temperature (A) and sieved particle size (C) were significant (*p* < 0.05), and the higher *F*-value of calcination temperature (A) and sieved particle size (C) terms in the linear effect indicated that the process of calcined shell powder preparation was greatly influenced by these two variables [25].

**Figure 1:**
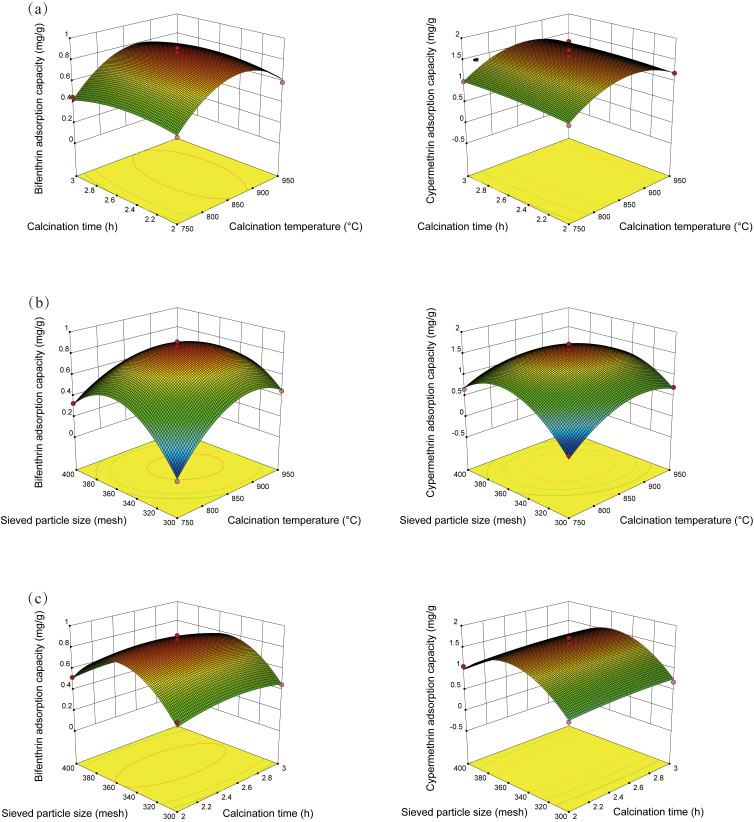
Response surface diagram for the combined effect of (**a**) calcination temperature and calcination time, (**b**) calcination temperature and sieved particle size, and (**c**) calcination time and sieved particle size on pyrethroid adsorption capacity.

**Figure 2:**
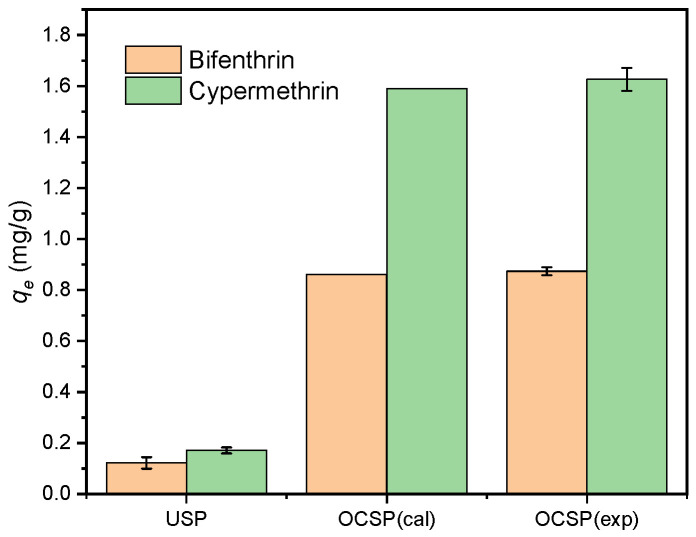
Adsorption of pyrethroids by different adsorbents (OCSP(cal) is calculated from the optimization model; OCSP(exp) is the experimental data).

The response surface diagram is shown in Figure 1, compared to calcination time, calcination temperature and sieved particle size are more significant in the pyrethroid adsorption model. The above results indicate that the calcination temperature and sieved particle size are important factors influencing the pyrethroid adsorption of OCSP; the calcination temperature being too low will lead to incomplete volatilization of impurities in the material and incomplete pore generation; conversely, too high calcination temperatures will lead to partial melting and slabbing of the material, collapse of existing pores, reduction of the specific surface area of the material, and deterioration of the adsorbent performance [26]. The influence of the sieved particle size on the adsorbent is reflected in the fact that the smaller the sieved size, the larger the specific surface area of the resulting adsorbent, and the more active adsorption sites per unit mass. However, when the sieved particle size of the adsorbent was too small, even after calcination, the volume of the material itself will limit the creation of pores, which was not conducive to the adsorption of the adsorbate [27,28]. In addition, the optimization experiment concluded the optimal conditions for the preparation of calcined shell powder (calcination temperature: 867 °C; calcination time: 2.49 h; and sieved particle size: 350 mesh). To validate the predicted optimal pesticide adsorption capacity given by the model, the OCSP under optimal conditions was made for pyrethroid adsorption, and the results (Figure 2) verify that the predicted capacity of pyrethroid adsorption by OCSP was generally consistent with the experimental result; OCSP showed a significant improvement in pyrethroid adsorption capacity compared to USP.

### 3.2. Characterization

#### 3.2.1. XRD Characterization

**Figure 3:**
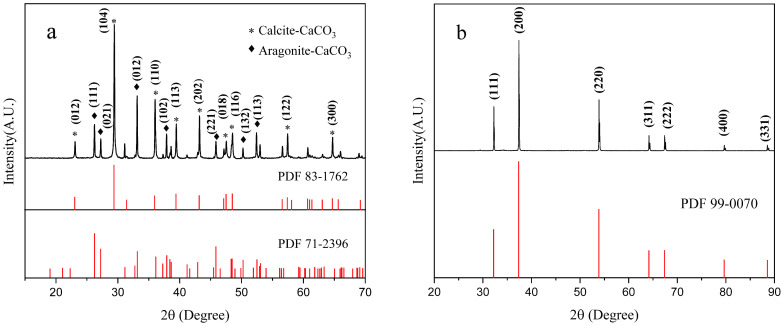
XRD of (**a**) USP and (**b**) OCSP.

Figure 3a,b show the XRD patterns of mussel shell powder before and after calcination, respectively. The diffraction peaks in Figure 3a are characteristic of calcium carbonate, in which the peaks at 29.41°, 36.00°, 39.44°, 43.19°, and 57.43° correspond to the main peaks of crystalline calcium carbonate calcite with Miller indices of (104), (110), (113), (202), and (122), respectively. The peaks at 26.22°, 33.12°, 37.87°, and 50.23° are the main peaks of the aragonite calcium carbonate, with corresponding Miller indices of (111), (012), (102), and (132), respectively. The diffraction peaks at 32.24°, 37.40°, and 53.89° in Figure 3b are the main diffraction peaks of CaO, corresponding to the Miller indices (111), (200), and (220), respectively. Comparing the physical phase analysis before and after calcination, only two crystalline forms of calcium carbonate were concluded to exist in the shell powder before calcination and no vaterite crystal form was found. This is because, compared to the other two crystalline forms, vaterite is very unstable under natural conditions; therefore, its diffraction peaks do not appear [29]. After calcination, the shell powder was completely transformed into calcium oxide [30], and its chemical structure and properties changed significantly.

#### 3.2.2. Micro Topography Analysis

**Figure 4:**
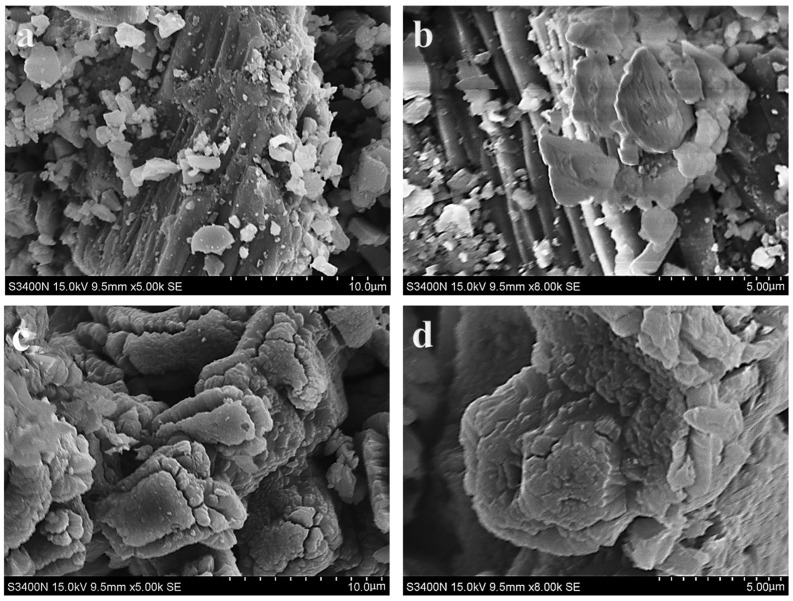
SEM images of (**a**,**b**) USP and (**c**,**d**) OCSP.

The adsorption capacity of an adsorbent is often related to its structure [31], and changes in the microscopic morphology of the sample before and after calcination can be observed using SEM. As shown in Figure 4a,b, the surface structure of the USP had a very dense lamellar structure, and only evident gully-like gaps existed between the layers, and the alignment of these gaps was basically in the same direction. In contrast, the surface of the OCSP shown in Figure 4c,d had very obvious cracks, which varied in size and had no obvious pattern in their distribution, indicating that a small amount of organic macromolecules, such as proteins and polysaccharides, existing in the original shell powder was completely lost after high-temperature calcination [32]. The rough surface and folded cracks of the calcined shell powder allowed better adsorption of environmental pollutants, which is one of the important reasons why inorganic mineral-based materials are often used as adsorbents.

#### 3.2.3. FT-IR Analysis

**Figure 5:**
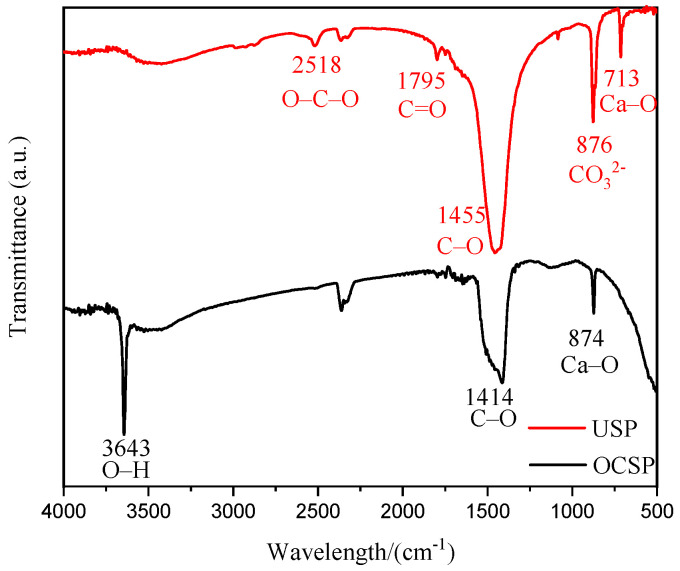
FT-IR analysis of USP and OCSP.

Figure 5 shows the FT-IR spectra of USP and OCSP. The USP peaks at 1455, 876, and 713 cm^−1^ showed obvious absorption peaks, where the absorption peak at 1455 cm^−1^ was typical for calcite with C–O asymmetric stretching vibration, and the peaks at 876 and 713 cm^−1^ were aragonite absorption peaks and calcite absorption peaks doped with a small amount of aragonite, respectively; the molecular vibration types corresponded to the CO_3_^2−^ out-of-plane bending vibration and Ca–O in-plane bending vibration, respectively [33,34]. In addition, there were three weak peaks in USP located at 2518, 1795, and 1080 cm^−1^, which were O–C–O asymmetric stretching vibration peaks in calcium carbonate, C=O bond [35], and symmetric stretching vibration in calcite mixed with a small amount of aragonite [36], respectively. Therefore, the main components of the shell powder before calcination were calcium carbonate, with calcite, and aragonite. There were also several distinct diffraction peaks of OCSP, with the sharpest and most elongated peak at 3643 cm^−1^, which is the characteristic peak of calcium oxide and is related to the O–H bond [37]. The peaks at 1414 and 874 cm^−1^ correspond to C–O bond and Ca–O bond [35]. Combined with the FT-IR analysis, the main composition of the shell powder before calcination was verified to be calcium carbonate containing calcite and aragonite crystals without vaterite crystals, while the shell powder after calcination at high temperature was completely converted to calcium oxide.

#### 3.2.4. BET and Pore Analysis

**Figure 6:**
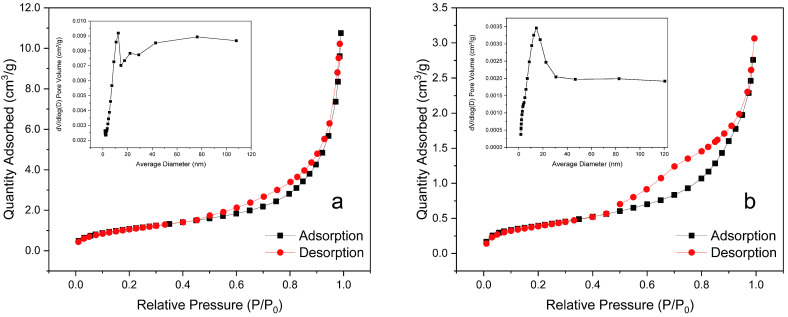
N_2_ adsorption–desorption isotherms and pore size distributions of (**a**) USP and (**b**) OCSP.

Figure 6 illustrates the nitrogen adsorption isotherms and pore size distributions of USP and OCSP. When P/P_0_ was higher than 0.4, the adsorption and desorption isotherms started to separate because of capillary condensation of the adsorbate, and the adsorption volume increased rapidly and produced a hysteresis loop, which is typical of type IV isotherm with type H3 hysteresis loop according to the IUPAC classification criteria and is commonly found in mesoporous materials [38,39]. Table 1 shows the specific surface area, pore volume, and pore size of USP and OCSP, and the pore sizes of the two materials were 12.80 and 15.48 nm, respectively, indicating that they were mesoporous materials. The specific surface area, pore volume, and pore size of the OCSP increased significantly, indicating that the impurities in the shell powder were calcined out after high-temperature calcination, and their original pores increased, which was more favorable for the adsorption of the adsorbate.

### 3.3. Effects of Reaction Time

**Figure 7:**
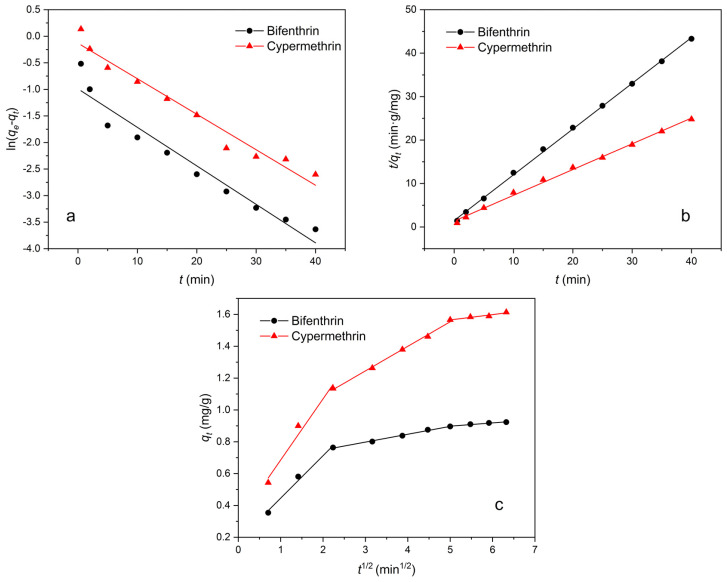
Pyrethroid adsorption capacity with (**a**) Pseudo first-order model, (**b**) Pseudo second-order model, and (**c**) Intra-particle diffusion model.

Figure 7 shows the kinetic models for the adsorption of pyrethroids at different reaction times, and the related parameters are shown in Table 2. As shown in Table 2, the pseudo second-order model has a higher R^2^ than other models, and the equilibrium adsorption capacity *q_e_* (cal) and *q_e_* (exp) of the pseudo second-order model are closer than the pseudo first-order model [12,40]. The data in the intra-particle diffusion (IPD) model suggest that the adsorption process is not a single linear process, and the rate constants of adsorption decrease in the order *Kp_1_*, *Kp_2_*, and *Kp_3_*, which reveals that the IPD is closely related to the adsorption process. The curves are not originated indicating that the IPD is not the capacity-limiting process to control the adsorption process [41], and the adsorption process may involve pesticides initially adsorbed on the surface of the adsorbent, then gradually spreading inward, and finally achieving dynamic equilibrium; even some chemical reactions are involved [42,43]. In summary, the adsorption of two pyrethroids on OCSP is more suitable to be described by the pseudo second-order and IPD model.

### 3.4. Adsorption Isotherms

**Figure 8:**
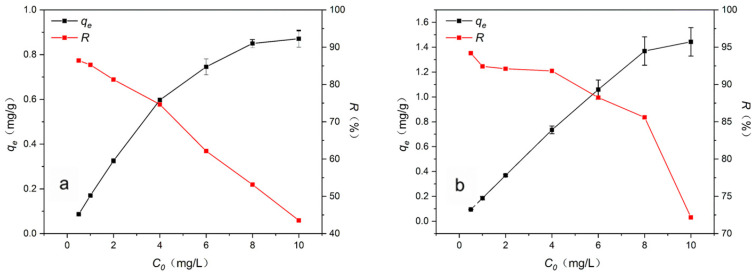
Effects of initial concentration of (**a**) bifenthrin and (**b**) cypermethrin on adsorption capacity and removal efficiency.

Figure 8 shows the effects of the initial concentration of pyrethroids on the adsorption capacity and removal efficiency; when the initial concentration of pyrethroids was 0.5 mg/L, the adsorption capacity was less than 0.1 mg/g, and the removal efficiency closed to 90%. As the initial concentration increased, the pesticide adsorption capacity increased, while the removal efficiency decreased. When the initial concentration of pesticides reaches the maximum, its adsorption capacity reaches the maximum at the same time, and the removal efficiency drops to the minimum. These results indicate that at low initial concentrations, the adsorbent has a large number of vacant sites that were not occupied, so there is a high pyrethroid removal efficiency and low adsorption capacity. With the increase in the initial concentration of pesticides, the adsorption capacity of pesticides also increased correspondingly with the continuous occupation of empty sites on the surface of the adsorbent, but gradually saturated adsorbent began to reduce the removal efficiency of pesticides. Finally, when the pesticide initial concentration reaches the maximum, the adsorption capacity reaches its highest value and the removal efficiency drops to its lowest value [44,45].

**Figure 9:**
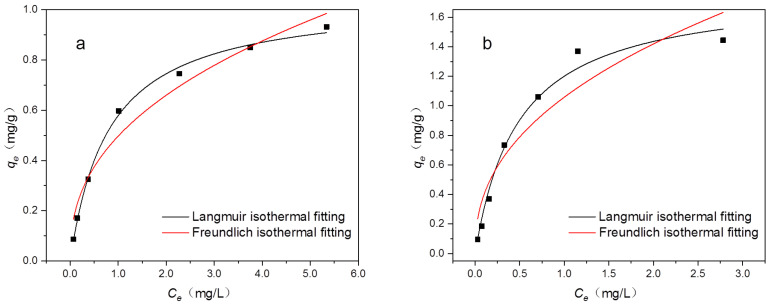
(**a**) Bifenthrin and (**b**) cypermethrin adsorption capacity with Langmuir isothermal fitting and Freundlich isothermal fitting.

Figure 9 and Table 3 show the results of fitting the Langmuir and Freundlich isotherms to the adsorption data and the corresponding model parameters. From the *q_m_* of the two pesticides derived from the Langmuir model, it can be concluded that OCSP has a high potential for pyrethroid adsorption; furthermore, by analyzing the *n* values of pyrethroids in the Freundlich model, it can be concluded that the adsorption process tends to be physical and the adsorbate is favorably adsorbed on the OCSP [26,46]. In summary, compared to the Freundlich model, the Langmuir model has higher R^2^ values, which is more suitable for describing the adsorption, and the adsorption process is mainly based on surface monolayer adsorption.

### 3.5. Effects of OCSP Dosages

**Figure 10:**
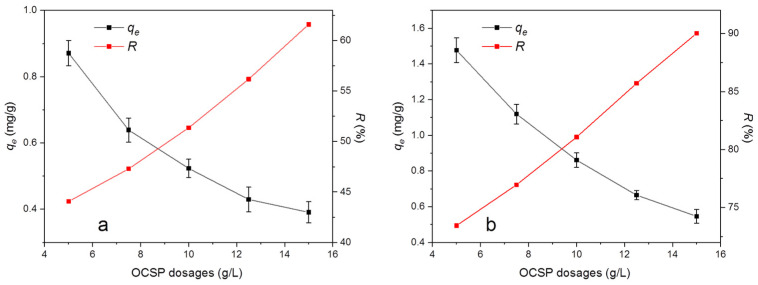
Effects of OCSP dosages of (**a**) bifenthrin and (**b**) cypermethrin on adsorption capacity and removal efficiency.

Figure 10 shows the effects of OCSP dosages on pyrethroid adsorption capacity and removal efficiency. With increasing dosages of OCSP, the adsorption capacity of both pyrethroids decreases continuously. The reason is that the adsorption site increases with the increase in the dosage of the adsorbent, but the total amount of adsorbate in the solution is constant and no more OCSP is needed, so the occupancy rate of the adsorption site contained in the unit mass of the adsorbent keeps decreasing, which leads to the constant decline in the adsorption capacity. Similarly, with the increase in the adsorbent dosage, the removal efficiency of the constant amount of adsorbate will increase continuously [47].

### 3.6. Effects of pH

Figure S2 shows the effects of pH on pyrethroid adsorption capacity. With the increase in the initial pH of the solution, the adsorption capacity of the adsorbent for the pyrethroid increased slightly; when pH reached 10.0, the adsorption capacity of the pyrethroid increased rapidly. This may be attributed to the fact that pyrethroids are stable under acidic conditions but unstable under alkaline conditions [48]. In addition, the results of zeta potential measurement showed that the zero charge (pH_pzc_) of the adsorbent was between 7 and 8, and the negative hydroxyl ions on the surface of the adsorbent increased when the pH was greater than pH_pzc_, which enhanced the electrostatic interaction between the negatively charged adsorbent and the pyrethroid molecules [49]. Therefore, the pH value of 10.0 is most suitable for pyrethroid adsorption.

### 3.7. Effects of NaCl Salinity

Figure S3 shows the effects of NaCl salinity on pyrethroid adsorption capacity. The adsorption capacity of pyrethroids by the adsorbent showed a decreasing trend with increasing salinity of NaCl in solution, which may be related to the increase in ionic strength leading to more Na^+^ competing with pyrethroids for adsorption sites through ion exchange [42]. In addition, similar studies have shown that an increase in NaCl concentration not only forms agglomerates that block the adsorption sites of the adsorbent [50], but also can affect the configuration and electrostatic nature of the adsorbate [51], which leads to a decrease in the adsorption of pollutants.

### 3.8. Reusability Studies

The results of the reusability study of the adsorbent are shown in Figure S4. With the increasing number of reuse, the adsorption capacity of pyrethroids by calcined shell powder decreased continuously, and when the number of reuses reached five, the decrease in adsorption performance was greater than before, but calcined shell powder still had a certain adsorption capacity of pyrethroids.

## 4. Conclusions

In this study, a high-efficiency absorbent was prepared from mussel shell powder, and the preparation process of OCSP was optimized using RSM. The calcination temperature and sieved particle size had a significant influence on its properties during the preparation process. The OCSP and USP characterization results demonstrated that after calcination, the main components of the structure changed from calcium carbonate to calcium oxide, and the specific surface area, pore volume, and pore space increased significantly. In addition, the mechanism of the adsorption process was studied, and the results showed that the Langmuir, pseudo second-order, and intra-particle diffusion models are more suitable for describing the adsorption process. In addition, the maximum adsorption capacity of bifenthrin and cypermethrin can reach 1.05 and 1.79 mg/g, and the removal efficiency is over 40 and 70% at the maximum initial concentration, respectively. In summary, OCSP is a promising and environmentally friendly adsorbent for pyrethroid adsorption in water.

## Figures and Tables

**Table 1:** Specific surface area, pore volume, and pore size of USP and CSP.

	BET Surface Area (m^2^/g)	Pore Volume (cm^2^/g)	Pore Size (nm)
USP	1.42	4.74 × 10^−3^	12.80
OCSP	3.97	1.66 × 10^−2^	15.48

**Table 2:** Adsorption kinetic model and the value of parameters.

Models and Parameters	Bifenthrin	Cypermethrin
Pseudo first-order		
*q_e_* (cal) (mg/g)	0.37	0.88
*k*_1_ (min^−1^)	0.17	0.15
R^2^	0.9444	0.9651
Pseudo second-order		
*q_e_* (cal) (mg/g)	0.95	1.69
*k*_2_ (g/mg·min)	0.75	0.26
R^2^	0.9994	0.9971
Intra-particle diffusion		
*k_p_*_1_ (mg/g·min^1/2^)	0.27	0.38
R^2^	0.9888	0.9743
*k_p_*_2_ (mg/g·min^1/2^)	0.05	0.15
R^2^	0.9941	0.9965
*k_p_*_3_ (mg/g·min^1/2^)	0.02	0.03
R^2^	0.9647	0.9383

The *q_e_* (exp) of bifenthrin and cypermethrin were 0.95 mg/g and 1.69 mg/g, respectively.

**Table 3:** Adsorption isotherm model and parameter values.

	Langmuir			Freundlich		
Pyrethroids	*q_m_* (mg/g)	*K_L_* (L/mg)	R^2^	*K_F_*	*n*	R^2^
Bifenthrin	1.05	1.24	0.9975	0.50	0.41	0.9641
Cypermethrin	1.79	2.05	0.9855	1.06	0.42	0.9002

## Data Availability

The data that support the findings of this study are available from the corresponding author upon reasonable request.

## Supplementary Materials

The following supporting information can be downloaded at: https://www.mdpi.com/article/10.3390/ma16072802/s1, Figure S1. Effect of (a) calcination temperature, (b) calcination time, and (c) sieved particle size on pyrethroid adsorption capacity. Different letters (a, b, c, d, and e) represent significant differences between different groups, *p* < 0.05. Figure S2. Effects of pH on pyrethroid adsorption capacity. Figure S3. Effects of NaCl salinity on pyrethroid adsorption capacity. Figure S4. Reusability study of adsorbent on pyrethroid adsorption capacity. Table S1. Factors and levels in the experiments. Table S2. Box–Behnken design of pyrethroids. adsorption capacity. Table S3. Analysis of variance (ANOVA) for response surface model of bifenthrin adsorption capacity. Table S4. Analysis of variance (ANOVA) for response surface model of cypermethrin adsorption capacity.
