# Supplementary material for: Adsorption of Pyrethroids in Water by Calcined Shell Powder: Preparation, Characterization, and Mechanistic Analysis
Source: Materials (Basel). 2023 Mar 31;16(7):2802. doi: 10.3390/ma16072802 (PMC10096194; doi:10.3390/ma16072802)
Supplement: Supplementary file 1 [file materials-16-02802-s001.zip › materials-2247180-supplementary.pdf]

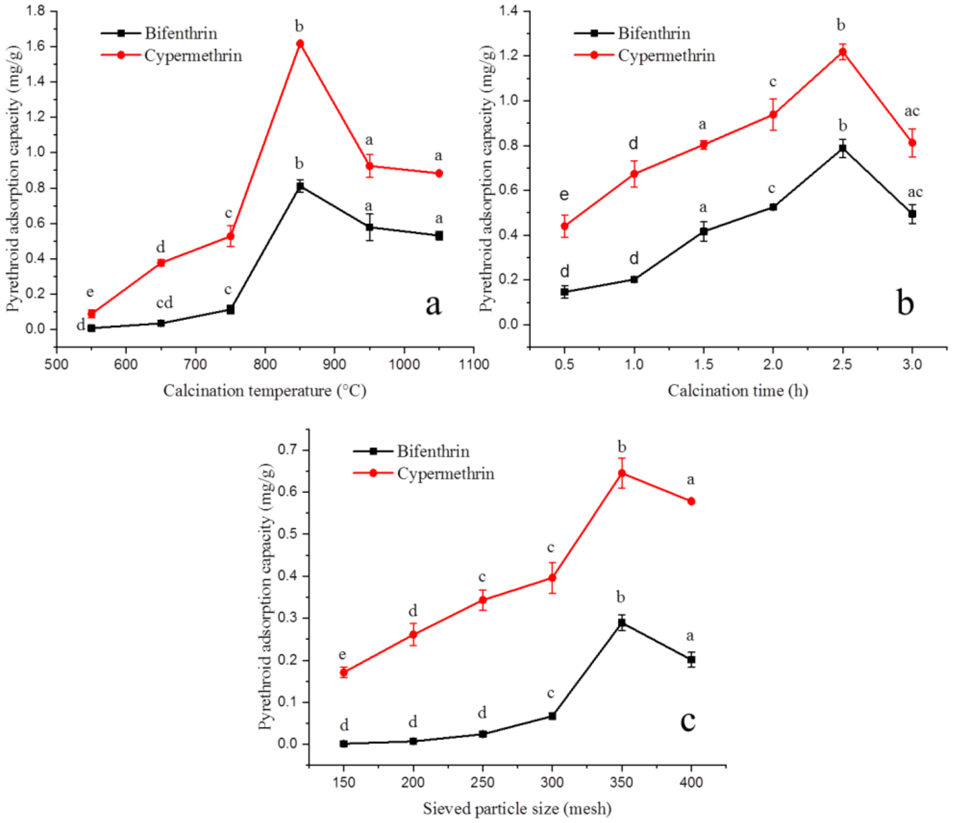

Figure S1. Effect of (a) calcination temperature, (b) calcination time, and (c) sieved particle size on pyrethroid adsorption capacity. Different letters (a, b, c, d, and e) represent significant differences between different groups, $p < 0.05$.

Table S1. Factors and levels in the experiments.

Factors	Code	-1	0	+1
Calcination temperature (°C)	A	750	850	950
Calcination time (h)	B	2	2.5	3
Sieved particle size (mesh)	C	300	350	400

Table S2. Box–Behnken design of pyrethroids. adsorption capacity.

Run order	A: Calcination temperature (°C)	B: Calcination time (h)	C: Sieved particle size (mesh)	Bifenthrin adsorption capacity (mg/g)	Cypermethrin adsorption capacity (mg/g)
1	1	1	0	0.65	1.29
2	0	-1	-1	0.46	0.66
3	0	0	0	0.91	1.58
4	-1	-1	0	0.44	0.85
5	0	1	1	0.42	1.11
6	0	0	0	0.83	1.49
7	0	-1	1	0.53	1.06
8	1	-1	0	0.59	1.19
9	0	0	0	0.82	1.52
10	-1	1	0	0.45	0.99
11	-1	0	-1	0	0.07
12	1	0	1	0.28	0.50
13	1	0	-1	0.45	0.71
14	0	0	0	0.87	1.48
15	0	1	-1	0.45	0.69
16	-1	0	1	0.33	0.67
17	0	0	0	0.87	1.72

Table S3. Analysis of variance (ANOVA) for response surface model of bifenthrin. adsorption capacity.

Source	Sum of Squares	df	Mean Square	F Value	p-value Prob > F
Model	0.98	9	0.11	65.11	< 0.0001
A-Calcination temperature	0.07	1	0.07	42.16	0.0003
B-Calcination time	2.0×10^{-4}	1	2.0×10^{-4}	0.12	0.7393
C- Sieved particle size	4.51×10^{-3}	1	4.51×10^{-3}	2.71	0.1440
AB	6.25×10^{-4}	1	6.25×10^{-4}	0.37	0.5598
AC	6.30×10^{-2}	1	6.30×10^{-2}	37.47	0.0005
BC	2.03×10^{-3}	1	2.03×10^{-3}	1.21	0.3070
A^2	0.29	1	0.29	173.95	< 0.0001
B^2	0.018	1	0.018	10.67	0.0138
C^2	0.47	1	0.47	279.10	< 0.0001
Residual	0.012	7	1.67×10^{-3}		
Lack of Fit	6.48×10^{-3}	3	2.16×10^{-3}	1.66	0.311
Pure Error	5.20×10^{-3}	4	1.30×10^{-3}		
Cor Total	0.99	16			

R^2＝0.9882　R^2 adj＝0.9730

Table S4. Analysis of variance (ANOVA) for response surface model of cypermethrin. adsorption capacity.

Source	Sum of Squares	df	Mean Square	*F* Value	*p*-value Prob > *F*
Model	3.20	9	0.36	35.58	< 0.0001
A-Calcination temperature	0.16	1	0.16	15.67	0.0055
B-Calcination time	1.30×10^{-2}	1	1.30×10^{-2}	1.28	0.2949
C- Sieved particle size	0.18	1	0.18	18.20	0.0037
AB	4.49×10^{-4}	1	4.49×10^{-4}	0.045	0.8382
AC	0.16	1	0.16	16.20	0.0050
BC	1.52×10^{-4}	1	1.52×10^{-4}	0.015	0.9053
A^2	0.80	1	0.80	79.72	< 0.0001
B^2	7.90×10^{-3}	1	7.90×10^{-3}	0.79	0.4037
C^2	1.72	1	1.72	171.56	< 0.0001
Residual	0.07	7	1.00×10^{-3}		
Lack of Fit	3.10×10^{-2}	3	0.01	1.03	0.4681
Pure Error	3.90×10^{-2}	4	9.86×10^{-3}		
Cor Total	3.27	16			

R^2＝0.9786 R^2 adj＝0.9511

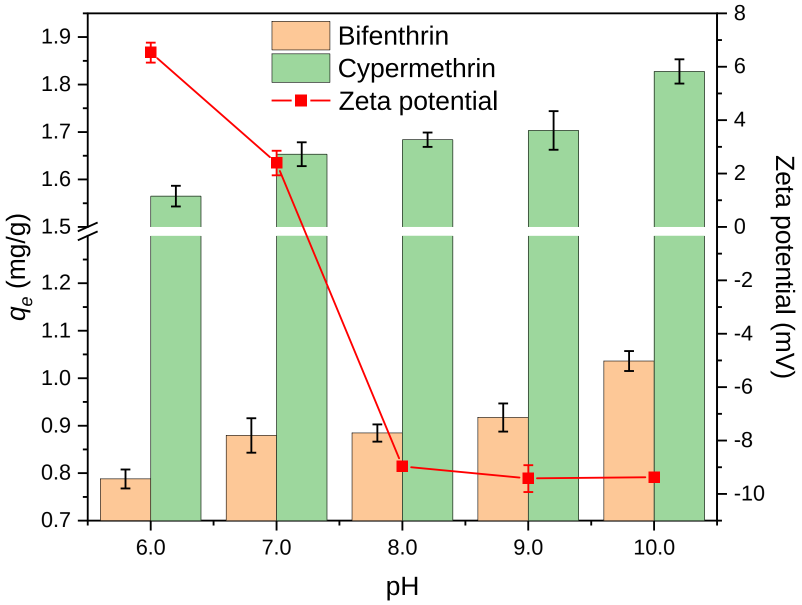

Figure S2. Effects of pH on pyrethroid adsorption capacity.

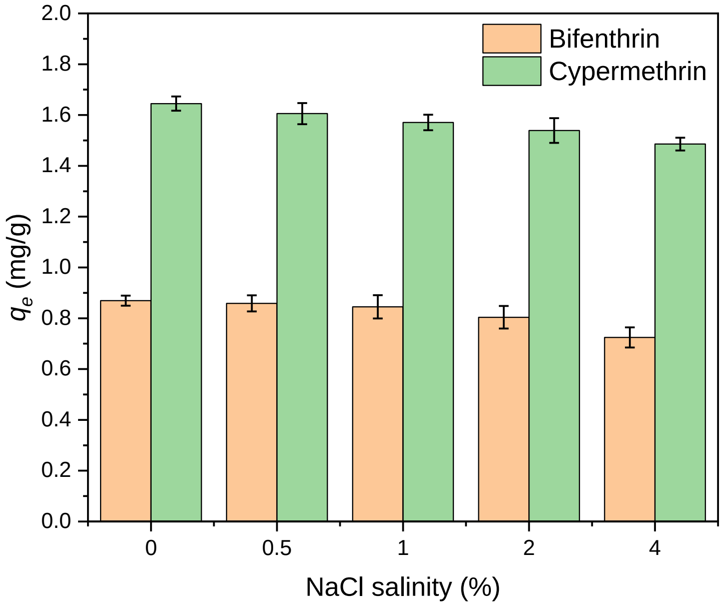

Figure S3. Effects of NaCl salinity on pyrethroid adsorption capacity.

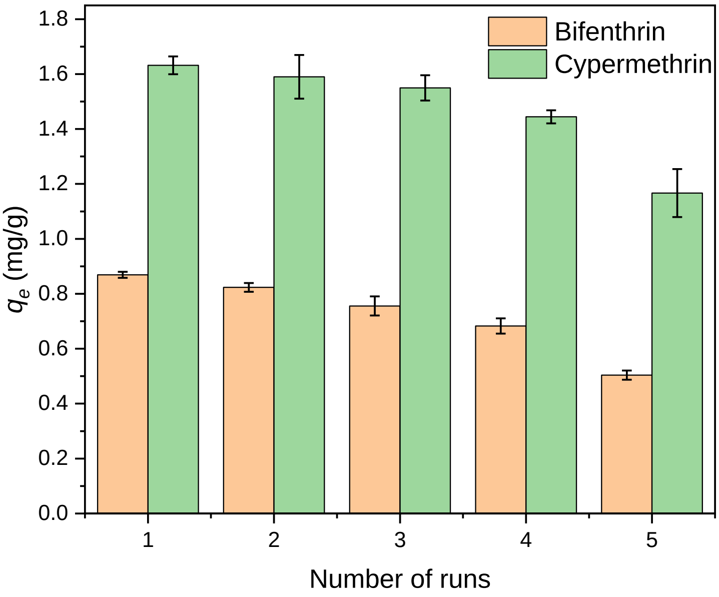

Figure S4. Reusability study of adsorbent on pyrethroid adsorption capacity.
